# Targeting the tumor stroma: integrative analysis reveal GATA2 and TORYAIP1 as novel prognostic targets in breast and ovarian cancer

**DOI:** 10.3906/biy-2010-39

**Published:** 2021-04-20

**Authors:** Ömer Faruk ERCEYLAN, Ayşe SAVAŞ, Esra GÖV

**Affiliations:** 1 Department of Bioengineering, Faculty of Engineering, Adana Alparslan Türkeş Science and Technology University, Adana Turkey

**Keywords:** Gene expression, cancer stroma, biomarkers, network medicine

## Abstract

Tumor stroma interaction is known to take a crucial role in cancer growth and progression. In the present study, it was performed gene expression analysis of stroma samples with ovarian and breast cancer through an integrative analysis framework to identify common critical biomolecules at multiomics levels. Gene expression datasets were statistically analyzed to identify common differentially expressed genes (DEGs) by comparing tumor stroma and normal stroma samples. The integrative analyses of DEGs indicated that there were 59 common core genes, which might be feasible to be potential marks for cancer stroma targeted strategies. Reporter molecules (i.e. receptor, transcription factors and miRNAs) were determined through a statistical test employing the hypergeometric probability density function. Afterward, the tumor microenvironment protein-protein interaction and the generic network were reconstructed by using identified reporter molecules and common core DEGs. Through a systems medicine approach, it was determined that hub biomolecules, AR, GATA2, miR-124, TOR1AIP1, ESR1, EGFR, STAT1, miR-192, GATA3, COL1A1, in tumor microenvironment generic network. These molecules were also identified as prognostic signatures in breast and ovarian tumor samples via survival analysis. According to literature searching, GATA2 and TORYAIP1 might represent potential biomarkers and candidate drug targets for the stroma targeted cancer therapy applications.

## 1. Introduction

Stroma is a mass of connective tissue that surrounds a set of cells formed by the elements involved in an organ or formation. Cancer is a disease involving multiple components of both tumor cells and stromal cells (Mao et al., 2013). Stromal cells participate in all steps of tumor initiation, progression, recurrence, metastasis and drug response, and finally, affect the prognosis of patients (Guo and Deng, 2018). Stromal cells in the microenvironment of the tumor have been shown to play an important role in cancer development. Molecular events in which active stromal cells affect cancer cells can be determined so that biomarkers and therapeutic targets can be identified (Valkenburg et al., 2018). Breast cancer is usually seen in the breast epithelium, but there is some important evidence that breast stromal cells also play an important role in tumor formation (Mao et al., 2013). Ovarian cancer, which proceeds from cell transformation through normal tissue invasion, is also connected to communication with the stromal microenvironment (Schauer et al., 2011). Both tumor types have common molecular characteristics. For example, BRCA1 and BRCA2 are common susceptibility genes for breast and ovarian cancer (King et al., 2003).

Advances in microarray and high-throughput sequencing technologies have provided effective applications to help develop more reliable biomarkers for diagnosis, survival and prognosis (Gov et al., 2017a). The predictive power of a single gene biomarker may be insufficient. The resulting studies have found that gene signatures, including several genes, may be better alternatives. The functions and mechanisms of gene signatures in diseases continue to be explored further. In several studies, the identification of molecular signatures to understand disease mechanism and explore the drug targets was studied such as key genes of three different ovarian diseases by using integrative systems biology analysis perspective (Kori et al., 2016), tissue-specific molecular biomolecules in ovarian cancer (Gov et al., 2017b), T2 diabetes (Calimlioglu et al., 2015), head and neck cancer (Islam et al., 2018) and Alzheimer disease (Rahman et al., 2020), as well as ovarian cancer stem cells (Gov, 2020). 

Cancer tissues produce special stroma, preferably for abnormal proliferation and invasion. Some other types of cells, such as fibroblasts, preexisting vascular cells, and mesenchymal stem cells, become potentially cancer-related fibroblasts (CAFs). Significant proinflammatory factors expressed by CAFs have been reported in some types of cancer such as breast and ovarian cancer (Erez et al., 2013). It was reported that infiltrated immune and inflammatory cells affect the molecular biology and clinical status of breast cancer (Karn et al., 2015). Planche et al. (2011) reported that the tumor microenvironment displays distinct features according to the cancer type that has prognostic predictive potentials in a study about the identification of common molecular signatures of breast and prostate tumor stroma.

In the present study, we performed an analysis of transcriptome datasets of ovarian cancer stroma and breast cancer stroma through an integrative systems biology perspective to identify common critical molecular signatures at multiomics levels. This study represents mutual reporter molecules for ovarian and breast cancer stroma as a potential prognostic molecular signatures and may provide a contribution about common cancer stroma response map for cancer treatment, diagnosis and prognosis.

## 2. Materials and methods

### 2.1. Selection of the gene expression datasets

The raw data of three transcriptome datasets related to breast cancer stroma [GSE26910 (Planche et al., 2011), GSE8977 (Karnoub et al., 2007) and GSE10797 (Casey et al., 2009)] and two datasets related to ovarian cancer stroma [GSE40595 (Yeung et al., 2013) and GSE38666 (Lili et al., 2013)] are obtained from Gene Expression Omnibus (GEO) (Barrett et al., 2013). The datasets originated from Affymetrix Human Genome U133 Plus 2.0 Array were selected. Samples of datasets were obtained from tumor stroma and normal stroma. A total of 79 tumor stroma and 43 normal stroma including 41 breast tumor stroma vs. 27 normal breast stroma and 38 ovarian tumor stroma vs.16 normal ovarian stroma were studied.

### 2.2. Identification of differentially expressed genes 

For identification of differentially expressed genes (DEG), CEL microarray raw data files were downloaded and affy package (Gautier et al., 2004) of the R (version 3.6) was employed. The executed dataset was normalized through robust multiarray (RMA) techniques (Bolstad et al., 2003) and linear models for microarray data (LIMMA) method (Smyth et al., 2003) were examined in the advanced statistical analysis of each dataset. DEGs were determined according to resultant p-values < 0.05 and fold changes were taken into account to determine the regulatory patterns of the DEGs (fold change >1.5 and <0.67).

### 2.3. Gene enrichment analysis of gene sets

Gene enrichment analysis was carried out via the ConsensusPathDB functional annotation tool (Kamburov et al., 2013) to determine the down and upregulated biological pathways statistically significant associated with DEGs. Reactome (Croft et al., 2011) and Kyoto Encyclopedia of Genes and Genomes (KEGG) (Kanehisa et al., 2012) were preferable used databases and enrichment results with a p-value of <0.01 were accepted. Each p-value is calculated utilizing the hypergeometric test for each of the biological pathways. The whole genome description and background for the human genome were utilized as the reference gene set.

### 2.4. Protein-protein interaction network reconstruction

The previously reconstructed comprehensive protein-protein interaction (PPI) network of Homo sapiens (Karagoz et al., 2016) which consists of 288,033 physical interactions between 21,052 proteins, was utilized. Cancer stroma-specific PPI network was reconstructed using the proteins encoded by the resultant core DEGs. In PPI networks, nodes represent proteins and edges represent interaction which is accepted as undirected between proteins. The networks were analyzed and visualized through Cytoscape (v3.6) (Smoot et al., 2011). To identify highly connected central proteins (i.e. hub protein) of PPI networks the dual-metric approach considering degree and betweenness centrality metrics simultaneously was used (Gov et al., 2017b). 

### 2.5. Identification of reporter molecules

To identify reporter molecules firstly interaction data were arranged. Interaction data were obtained from our previous study (Comertpay and Gov, 2020) consisting of 284 TFs, 2599 miRNAs, 916 receptors and 22808 genes. Reporter molecules were determined via employing the hypergeometric probability density function by using the physical interaction of TFs, miRNAs and receptors with core DEGs obtained from breast and ovarian cancer stroma datasets.

The adopted our procedure (Comertpay and Gov, 2020) was applied to gene expression data of cancer stroma and employed in the prediction of molecular signature in the tumor microenvironment. Reporter molecules were identified according to computed p-values < 0.05.

### 2.6. Tumor microenvironment generic network reconstruction

The reconstruction of stroma specific network was employed by using reporter receptors, regulatory reporter molecules, TFs and miRNAs, interacted with target core DEGs. The visualization of network was provided via Cytoscape (v3.6) which is an open-source software platform (Smoot et al., 2011).

### 2.7. Prognostic performance analysis

Cox survival analysis was performed to determine the prognostic performance of the hub genes in the tumor microenvironment generic network using comprehensive microarray and RNA-Seq datasets. In the analyses, breast cancer dataset (n = 962) from TCGA and ovarian cancer dataset (n = 329) from TCGA and ICGC databases were employed. Cox proportional hazards regression analysis was executed through the SurvExpress validation tool (Aguirre-Gamboa et al., 2013). In SurvExpress, the cancer samples were grouped into low- and high-risk groups according to their prognostic index calculated using patient survival times. The prognostic capabilities of the hub genes were identified through Kaplan–Meier plots and the log-rank test. Furthermore, heat map representation was used to the gene expression pattern of hub genes according to low- and high-risk groups and the p-value was obtained from the Student’s t-test.

Cox-survival analysis of hub miRNAs was performed by using the Kaplan–Meier plotter tool (Nagy et al., 2018). In the pan-cancer section, breast cancer (n = 1077) and ovarian cancer (n = 486) datasets were used for drawing of Kaplan–Meier plots.

## 3. Results

### 3.1. Mutual tumor microenvironment signatures for breast and ovarian cancer 

**Figure 1:**
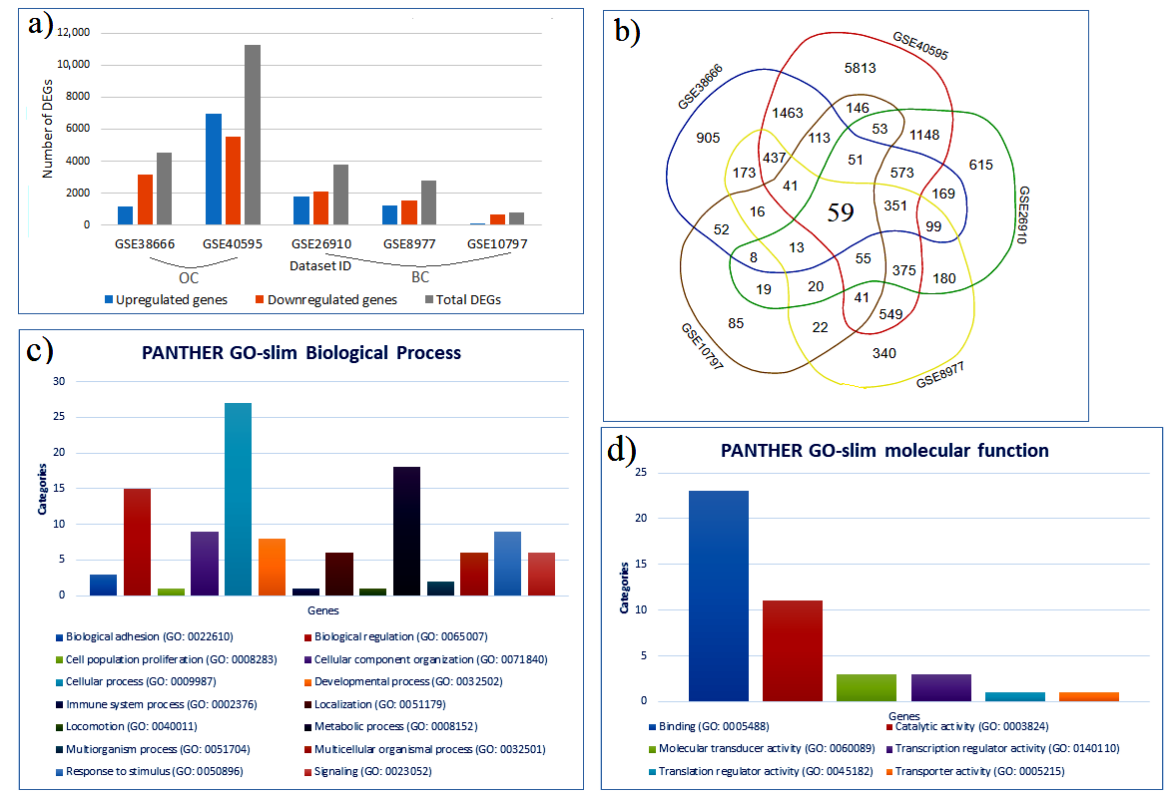
Differentially expressed genes (DEGs) in breast and ovarian tumor stroma. a) The distribution of DEGs in stromal cells of breast and ovarian cancer. Downregulation and upregulation of DEGs were represented by orange and blue colors, respectively. b) Venn diagram representation for the comparison of DEGs among stromal cells of breast and ovarian cancer. Core DEGs in all samples. c) PANTHER classification of core DEGs according to their biological process. d) PANTHER classification of core DEGs according to their molecular function.

The microarray datasets obtained from the stroma of breast and ovarian cancer were analyzed. For both cancer stroma gene expression analyses, it was determined that the number of the downregulated genes is a little higher than the upregulated genes number except GSE40595 (Figure 1a). The integrative analyses of DEGs indicated that there were 59 mutual core DEGs were identified between the five datasets (Figure 1b). These core DEGs were considered as mutual tumor stroma signatures for breast and ovarian cancer. The genes were classified according to their molecular functions and biological processes. It was determined prominent biological processes like the cellular process (45.7%), metabolic process (30%) and biological regulation (25.4%) (Figure 1c) and molecular activities as binding (39%), catalytic activity (18.6%) (Figure 1d). PANTHER GO-slim analysis showed that the core DEGs were enriched in different biological processes and molecular functions.

**Figure 2:**
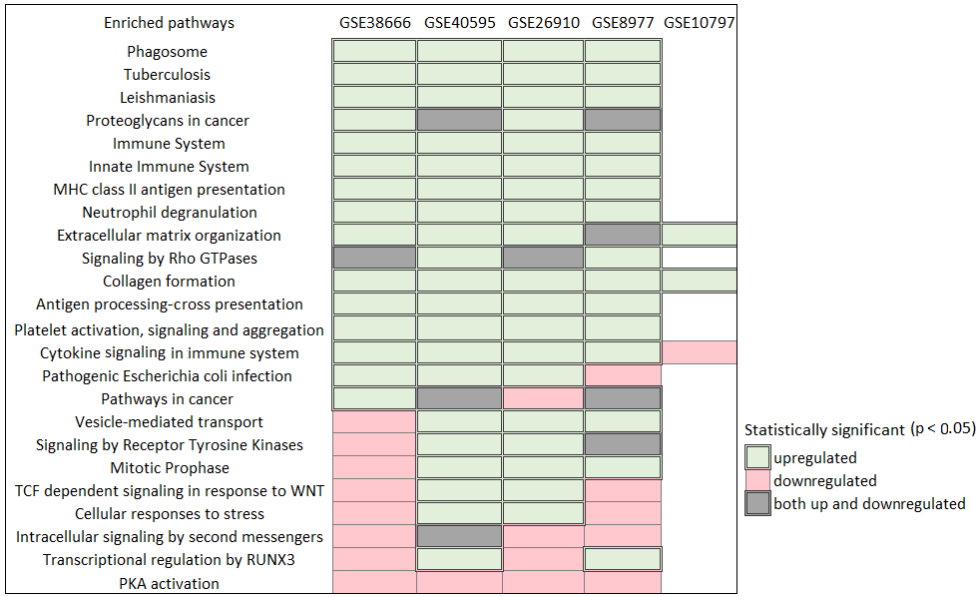
Statistically significant biological pathways in each datasets containing breast and ovarian cancer stroma samples. Downand upregulated gene list of each dataset was used to obtain down- and up-egulated pathways. Downregulation and upregulation of pathways were represented by pink and green colors, respectively. Both up- and downregulated pathways were represented by grey color.

The biological pathway enrichment analysis of each dataset revealed that common pathways in immune systems related pathways (37.5%), signaling pathways (29%), and stroma associated pathways such as proteoglycan in cancer, collagen formation, extracellular matrix organizations were altered (Figure 2). It was determined that common pathways mostly upregulated (green colors in Figure 2) while PKA activation is downregulated in four datasets. Common enriched pathways of GSE10792 is low due to it has few number of DEGs compared to other datasets. 

### 3.2. Tumor microenvironment protein interaction network 

The first neighbor enriched PPI network of the proteins of corresponding mutual core DEGs was constructed including 907 nodes and 1660 edges. Tumor stroma specific PPI network represented scale-free topology with a few highly-connected proteins. The hub proteins including EGFR, STAT1, VDR, NCOA1, CTBP2, MET, EIF3B, LEF1, KIF1B and CIRBP were identified by using degree (local-based) and betweenness centrality (global-based) metrics (Kori et al., 2016).

### 3.3. Tumor microenvironment generic network with enriched reporter biomolecules

**Figure 3:**
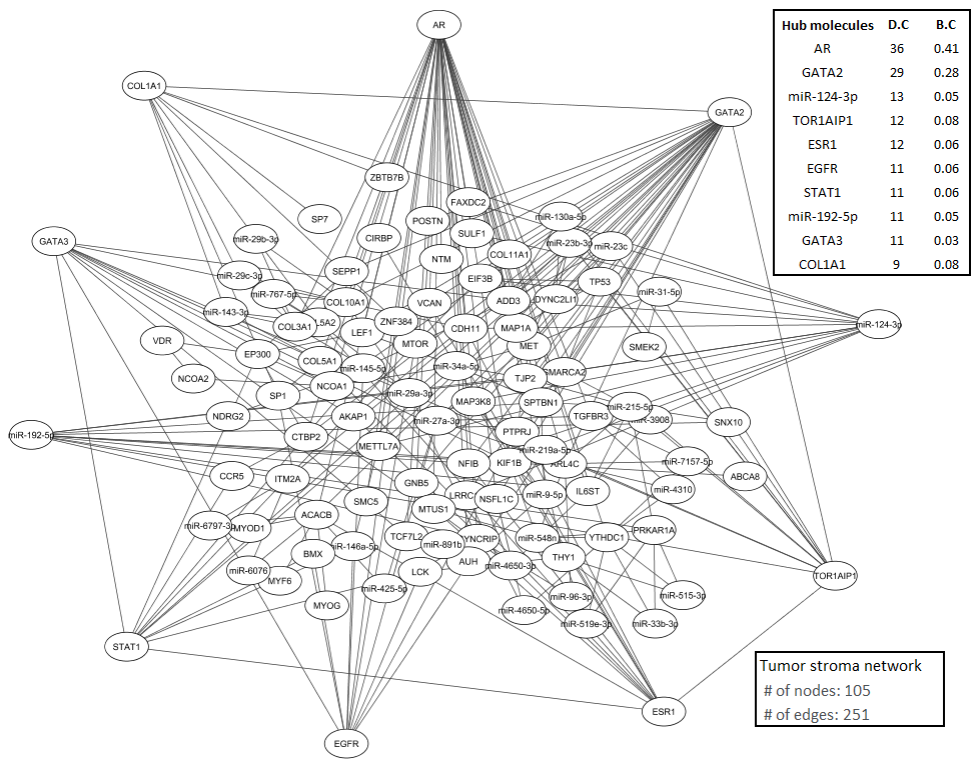
Reconstructed tumor microenvironment generic network and hub biomolecules.

To identify common breast and ovarian cancer stroma response map was constructed using core DEGs and reporter biomolecules. According to hypergeometric probability analysis results, 7, 12 and 32 reporter receptors, TFs and miRNAs significantly interacted with core DEGs were identified (p < 0.01), respectively (Table 1). The results have been mapped by using core DEGs reporter biomolecules interaction and it was reconstructed tumor microenvironment generic network of breast and ovarian cancer including 105 nodes and 251 edges (Figure 3). Through topological analysis, hub biomolecules (AR, COL1A1, EGFR, ESR1, GATA2, GATA3, miR-124-3p, miR-192-5p, STAT1 and TOR1AIP1) were identified. Interestingly, COL1A1 and TOR1AIP1 which are mutual core DEGs were determined as highly connected with reporter biomolecules. On the other hand, EGFR and STAT1 are both core DEGs and hub proteins in the PPI network. The rest of the reporter biomolecules are TFs and miRNAs having transcriptional regulatory and posttranscriptional regulatory functions. 

**Table 1:** Reporter molecules in the mutual tumor stroma of breast and ovarian cancer.

	p-value	Reporter TFs	p-value	Reporter receptors	p-value
miR-192-5p	3.00E-05	AR	2.17E-07	BMX	0.0032
miR-124-3p	4.50E-05	ESR1	9.35E-05	CCR5	0.0038
miR-33b-3p	0.0001	GATA2	0.0055	EP300	0.0006
miR-519e-3p	0.0002	GATA3	0.0002	LCK	0.0052
miR-515-3p	0.0002	MYF6	0.0025	MTOR	0.0007
miR-145-5p	0.0002	MYOD1	0.0025	NCOA2	0.0097
miR-548n	0.0004	MYOG	0.0025	PTPRJ	0.004
miR-27a-3p	0.0004	SP1	0.0067		
miR-4650-5p	0.0005	SP7	0.0025		
miR-215-5p	0.0005	TP53	0.0016		
miR-143-3p	0.0007	ZBTB7B	0.0051		
miR-9-5p	0.001	ZNF384	0.0051		

**Figure 4:**
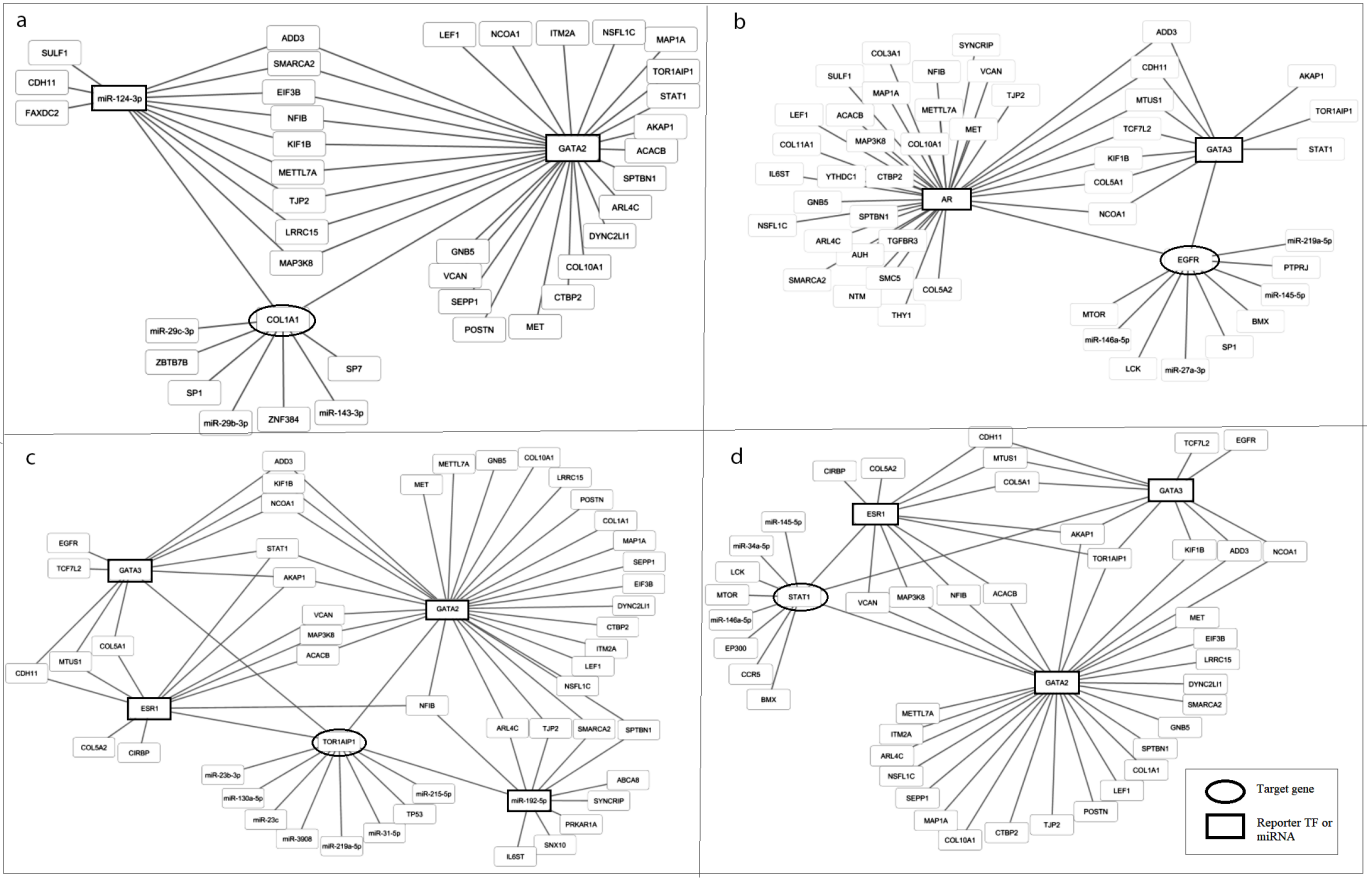
Scenarios of interactions among mutual hub biomolecules in breast and ovarian tumor stroma. a) interaction of transcriptional regulators and core DEG, b) interaction of transcriptional regulators and both hub protein in PPI network and core DEG, c) interaction of transcriptional regulators and core DEG, d) interaction of transcriptional regulators and both hub protein in PPI network and core DEG.

From a generic network, it was revealed resultant reporter biomolecules interact within themselves. We obtain some of the scenarios (Figure 4) such as regulatory biomolecules GATA2 and miR-124-3p interacted with COL1A1 (Figure 4a). It was determined the interaction of AR, GATA3 as a reporter TFs and EGFR which is both a hub protein in the PPI and a core DEG (Figure 4b). In the other interesting scenario, TOR1AIP1 interacts with ESR1, GATA2, GATA3 which are reporter TFs and miR-192-5p (Figure 4c). Similarly, ESR1, GATA2 and GATA3 interact with STAT1 which is both a hub protein in the PPI and a core DEG (Figure 4d). Table 2 represents description of hub biomolecules using by GeneCards database (Safran et al., 2010). It was suggested that these ten hub biomolecules may be significant novel molecular signatures in stroma targeted cancer treatment.

**Table 2:** The biological importance and descriptions of hub biomolecules in the present study.

Hub biomolecules	Biological importance
AR	The protein function of AR is a steroid-hormone activated TF that affect proliferation and differentiation in target tissues.
GATA2	GATA2 is a TF involved in stem cell maintenance with important roles in hematopoietic development.
miR-124	Among its related pathways are MicroRNAs in cancer and Alzheimers Disease.
TOR1AIP1	The protein of this gene is responsible for nuclear membrane integrity.
ESR1	Nuclear hormone receptor. ESR1 is clinically relevant in breast, endometrial, ovarian and other cancer types.
EGFR	It is a transmembrane glycoprotein and its amplification and mutations have been shown to be driving events in many cancer types.
STAT1	Signal transducer and TF that mediates cellular responses to interferons, cytokines and growth factors.
miR-192	Among its related pathways are MicroRNAs in cancer.
GATA3	GATA3 is a TF and an important regulator of T-cell development. It is required for the T-helper 2 differentiation process in the immune response.
COL1A1	It is a fibril-forming collagen found in most connective tissues.

### 3.4. Potential prognostic targets in the tumor microenvironment

**Figure 5:**
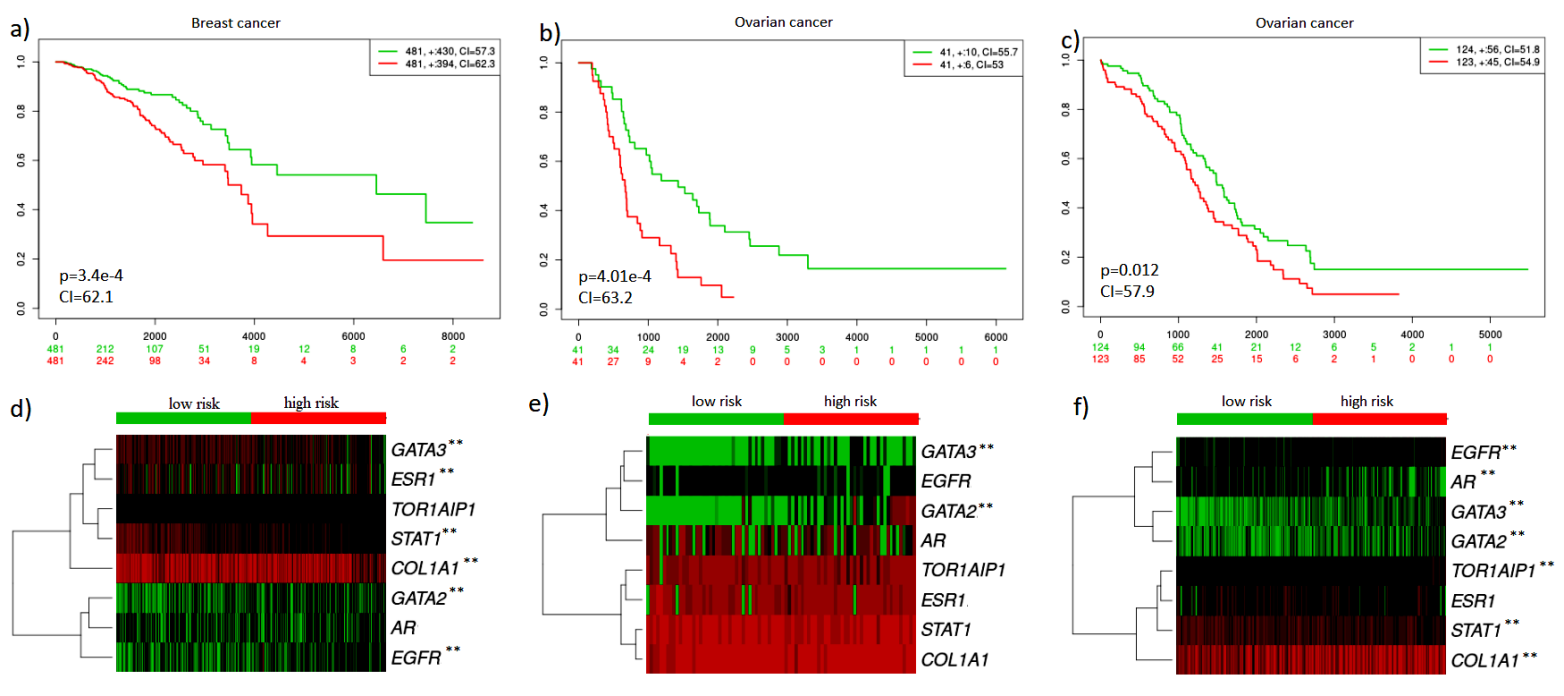
Prognostic potential of the hub biomolecules in the tumor microenvironment generic network. a) Kaplan–Meier plot for breast cancer patients obtained from the TCGA database, b) Kaplan–Meier plot for ovarian cancer patients obtained from the ICGC database, c) Kaplan–Meier plot for ovarian cancer patients obtained from the TCGA database. The p-values are computed via the longrank test (p < 0.05). Heat map represents the expression of hub genes (rows) along with samples (columns) in risk groups for d) breast cancer samples, e) ovarian cancer samples obtained from ICGC database, f) ovarian cancer samples obtained from TCGA database. The green and red grades represent the downregulated and upregulated expression, respectively. Two stars (**) marks genes represent p-value <0.05 and no stars represent p-value is >0.05. The difference of gene expression between risk groups compare using a t-test were presented by box plots.

**Figure 6:**
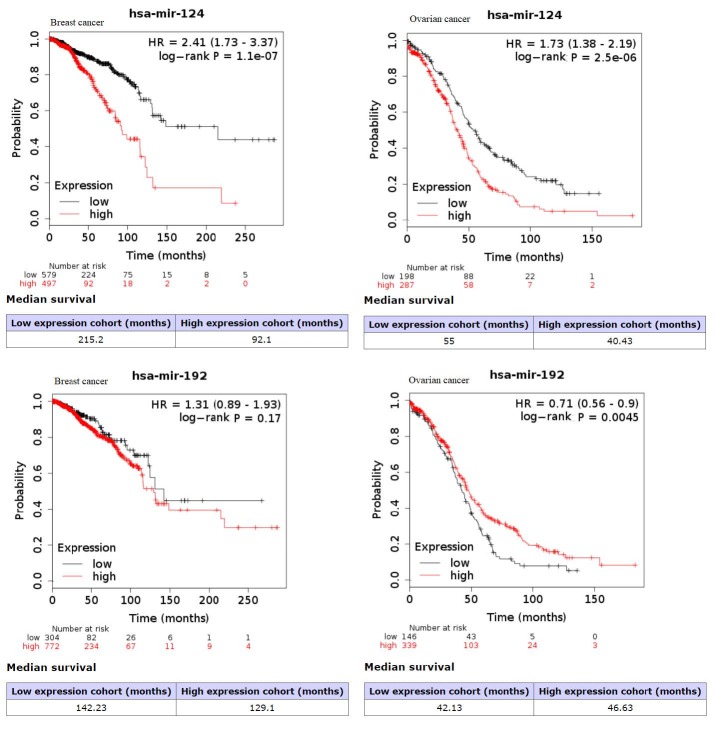
Kaplan–Meier graphs of hub miRNAs in the tumor microenvironment generic network according to KM plotter tool result (p < 0.05).

Stromal content of TCGA datasets of breast and ovarian cancer were identified via Estimation of STromal and Immune cells in MAlignant Tumours using Expression (ESTIMATE) data method. According to the results, the relatively high stromal score was found in breast carcinoma and high-grade serous ovarian carcinoma (Yoshihara et al., 2013). Kaplan–Meier graphs for estimating the survival curve, the log-rank test to compare two groups statistically were used to determine the prognostic potential of hub genes in the generic network. According to the analysis, statistically significant results could be obtained for all datasets (Figures 5a–5c). Furthermore, gene expression profiles of the hub genes were represented via heat maps (Figures 5d–5f). ESR1, TOR1AIP1, STAT1, COL1A1 were identified as high expression, while EGFR, AR, GATA2 were identified as a low expression in both cancer types. Only GATA3 represented different expression profiles including the high expression for breast cancer (Figure 5d) and low expression for ovarian cancer (Figures 5e and 5f). Kaplan–Meier graphs of hub miRNAs, miR-124 and miR-192 were also represented (Figure 6). Although miR-124 was identified as prognostic miRNAs for both cancer type, miR-192 was determined as prognostic biomolecule for only ovarian cancer, it was not obtained statistically significant result for breast cancer. It was suggested that hub biomolecules of the tumor microenvironment generic network obtained from breast and ovarian cancer stroma samples represent prognostic biomolecule potentials in patients with breast and ovarian cancer. 

## 4. Discussion

Understanding of the pathogenic mechanism and molecular signatures of tumor stroma should provide valuable insight into cancer initiation and progression. We studied differential gene expression in breast and ovarian cancer stroma with the common regulatory patterns, common key pathways, cancer stroma associated PPI and tumor microenvironment generic network to identify central molecular signatures that may serve as potential prognostic or therapeutic targets in breast and ovarian cancer. The difference of this study from other current studies is to analyze the ovarian cancer stroma and breast cancer stroma datasets separately, then to identify the common molecular signatures, to determine the common network structures and to discover some stromal features that can guide the prognosis and treatment. These features can give a holistic view of breast cancer stroma and ovarian cancer stroma, as well as the hub biomolecules obtained, which can be used as guiding target molecules in prognostic and therapeutic applications. 

Analyzing the gene expression patterns of each dataset were enriched common pathways in immune systems related pathways, signaling pathways, and stroma associated pathways such as proteoglycan in cancer, collagen formation, extracellular matrix organizations. Moreover, the core DEGs were enriched in different biological processes and molecular functions such as binding and catalytic activity. PPI network provides a comprehensive framework for exploring the basic mechanisms behind human disease (Sevimoglu and Arga, 2014). The cancer stroma specific network was reconstructed by using reporter receptors, regulatory reporter molecules, TFs and miRNAs, interacted with target core DEGs (Table 1). Through a holistic approach, it was determined that hub biomolecules of tumor microenvironment generic network and hub proteins of PPI network interact within themselves. These hub biomolecules of tumor stroma network, AR, GATA2, miR-124, TOR1AIP1, ESR1, EGFR, STAT1, miR-192, GATA3, COL1A1, may give crucial information about tumorigenesis (Table 2). Because stroma associated cells and factors have a supportive role in carcinogenesis they are expressed during cancer initiation and progression (Bhowmick and Moses, 2005). Thus, altered and highly interacted hub biomolecules may provide key information on the dysregulation of gene expression in the carcinogenesis of ovarian and breast tissues. Moreover, the resultant biomolecules were also identified as prognostic biomolecules in the tumor samples. 

Prognostic stroma-related genes were subject to literature data mining in terms of association with tumor stroma and malignancies. Henshall et al. (2001) reported that AR expression in tumor epithelium and stroma that is associated with a poor clinical outcome in prostate cancer, on the other hand, AR is emerging as a potential new therapeutic target for the treatment of breast cancer (Giovannelli et al., 2018). Epidemiological and preclinical studies have been made showing the crucial potential involvement of AR signaling in ovarian tumorigenesis (Mizushima and Miyamoto, 2019). GATA2 gene has been identified in stroma-related studies in colon cancer prognosis (Uddin et al., 2019), and also reported as a molecular signature in ovarian cancer via network medicine perspective (Gov et al., 2017). Wang et al. (2016) reported that miR-124-3p is a tumor suppressor in breast cancer. Various research has shown that miR‐124 may act as a tumor suppressive by regulating different target genes in several cancers such as prostate cancer (Shi et al., 2013), and head and neck cancer (Zhao et al., 2017). It is widely known that the use of inhibitors of ER (ESR1) in the treatment of patients with estrogen-positive breast cancer has offered a good prognosis (Tong et al., 2018). Moreover, the ESR1 gene is frequently methylated in many types of gynecological malignancies such as highly expressed in epithelial ovarian cancer (Giannopoulou et al., 2018). EGFR is the other well-known cancer-related protein. Wang et al. (2016) recently reported that high expression of EGFR in tumor stroma has a correlation with aggressive clinical properties in epithelial ovarian cancer, and is a prognostic factor. On the other hand, that upregulated expression of EGFR protein has been reported to occur in 16%–36% of breast cancers (Bhargava et al., 2005). Zellmer et al. (2017) showed that STAT1 expression in stroma promotes tumor progression and it is a potential target for breast cancer treatment. STAT1 is a tumor suppressor gene in breast cancer (Koromilas and Sexl, 2013) and upregulated STAT1 expression with better response to chemotherapy in patients with ovarian cancer (Josahkian et al., 2018). Hu et al. (2013) reported that miR-192 expression is significantly downregulated in breast cancer tissue and the miR-192/215 family is upregulated in mucinous ovarian tumor samples (Agostini et al., 2018). GATA3 takes a crucial role in normal mammary gland development, and its expression demonstrates high correlation with the estrogen receptor α (ERa) in human breast tumors (Eeckhoute et al., 2007). Moreover, it was showed that GATA3 expression is related to poor prognosis of high-grade serous ovarian carcinoma patients (Chen et al., 2018). Recently it was reported that COL1A1 secreted by fibroblasts promoted stromal cells and facilitates the metastasis of ovarian cancer, which may provide a novel approach for ovarian cancer therapeutics (Li et al., 2020).

Considering the potential role of identified molecular signatures in the tumor microenvironment, two biomolecules, GATA2 and TORYAIP1, might be a novel candidate for the treatment in the breast and ovarian cancer. To the best of our knowledge there has been no published report that explained they would be utilized as a novel candidate for molecular signatures in tumor stroma and the treatment of these cancers. We suggest experimental studies to identify the possible role of these proteins. The present study shares a novel approach regarding the molecular mechanism and identification of potential molecular signatures and candidate drug targets for the stroma targeted cancer therapy applications. 
